# Regulation of Shootin1 Gene Expression Involves NGF-induced Alternative Splicing during Neuronal Differentiation of PC12 Cells

**DOI:** 10.1038/srep17931

**Published:** 2015-12-09

**Authors:** Volkan Ergin, Mutlu Erdogan, Adnan Menevse

**Affiliations:** 1 Department of Medical Biology & Genetics, Faculty of Medicine, Gazi University, 06560 Ankara, Turkey; 2 UNAM - Institute of Materials Science and Nanotechnology, Bilkent University, 06800 Ankara, Turkey

## Abstract

Shootin1 is a protein involved in neuronal polarization, and has been shown to be a key molecule for the positive/negative feedback loop for axon induction required during neuronal symmetry breaking. To better understand the molecular basis of shootin1 dynamics, we analysed the regulatory pathways and the expressional status of shootin1 gene during NGF-induced neuronal differentiation. We demonstrated that the isoform-1 and isoform-2 of shootin1 is differentially expressed during neuronal differentiation. By blocking individual downstream pathways of NGF signalling, we found that PI3K/Akt pathway plays a major role in the expression of shootin1 isoform-2. Western blot and RT-PCR results showed that the isoform-1 of shootin1 is constitutively expressed, while the isoform-2 is expressed in a manner that is strictly dependent on NGF-stimulation. Isoform-specific RT-PCR results demonstrated that the differential expression of the isoform-1 and isoform-2 of shootin1 is a consequence of alternative splicing of shootin1 pre-mRNA, in response to NGF-signalling. Collectively these findings provide the first information on the molecular mechanisms regulating the expression of shootin1 gene and represent the first example of NGF-induced alternative splicing process that has a regulatory role in neuritogenesis.

Neuronal polarity refers to the asymmetric organization of neuronal compartments and structures, and essentially manifests the basic morphology of neurons characterized with a single axon and multiple dendrites^1^. The molecular dynamics underlying the neuronal polarization processes have been under investigation for decades, on the other hand, the mechanisms and mediators of intracellular polarization signals and how these internal signals emerge in the presence or absence of external asymmetric cues are still in question^2^.

A novel protein named shootin1, which was originally described as a brain-specific protein, has recently been identified as a potential mediator of axon formation by asymmetrically accumulating in a single, early-stage neurite, which then leads to neuronal polarization. Overexpression of shootin1 leads to the generation of surplus axons, and siRNA knockdown hinders axon formation. Shootin1 was also shown to colocalize with active pools of phosphoinositide 3-kinase (PI3K) and inhibition of PI3K activity reduces the ability of shootin1 to induce surplus axons^3^. Recent findings demonstrated that shootin1 is a novel KIF20B cargo and the interaction with this member of the kinesin superfamily localizes shootin1 to the tip of the elongating axon, in addition to that, shootin1 seems to interact with PI3K so that this interaction leads to local phosphatidylinositol (3,4,5)-trisphosphate (PIP3) activity in the growth cone^4^. Another recent study showed that p21-activated kinase 1 (Pak1), which is a downstream effector of Cdc42 and Rac1, mediates the phosphorylation of shootin1, thus promotes the clutch engagement and transmission of traction forces at axonal growth cones^5^.

In addition to these results, quantitative live cell imaging studies and modelling analyses showed that neuronal anterograde transport and retrograde diffusion of shootin1 generates an asymmetric signal for axonogenesis, which leads to a mechanism that enables neurite length‐sensing involved in neuronal symmetry breaking^6^.

In spite of these findings regarding the function of shootin1 protein, there is no experimental data on the genetic regulation of the shootin1 gene. Bioinformatic analysis of the putative promoter sequence of shootin1 gene indicated that the transcription of the gene seems to be regulated by a TATA-less, GC-rich promoter, which are typical promoter properties of neuronal genes^7^. Shootin1 gene (KIAA1598; *SHTN1*; GenBank ID: 57698; GRCh38 r.106) is located on 10q25.3 in human and consists of 243210 nucleotides; it has 5 transcript variants named as isoform a, b, c, d and e and they encode for 631, 456, 571, 558 and 498 amino acid-proteins, respectively. However, there is not yet functional data provided for any of the variants other than the 456 amino acid-isoform (Shootin1 isoform-2) expressed in rat. Rat shootin1 gene (RGD1311558; *Shtn1*; GenBank ID: 292139; Rnor_5.0 r.104), which is located on 1q55, consists of 104223 nucleotides and spans 17 exons. There are two transcript variants in rat named as Shootin1 isoform-1 (NCBI RefSeq: NM_001079705.3) and as Shootin1 isoform-2 (GenBank: EF055485.1), which encode for 3581 and 1371 nucleotides, respectively; these transcripts are translated into 633 and 456 amino acids, respectively. Both isoforms contain three coiled-coil domains, together with a proline-rich region. Shootin1 protein does not exhibit significant homology to previously known polypeptides, which suggests that it is a member of a novel class of proteins, and because of the lack of an invertebrate homologue in databases, shootin1 seems to have emerged lately in the genome of animals during evolution^3^.

Although there are not yet experimentally confirmed functional data on the isoform-1, it has previously been demonstrated by Toriyama *et al.* that the expression level of shootin1 isoform-2 is significantly increased at stage 2/3 transition during polarization of rat hippocampal neurons, and via immunoblot analyses of various rat tissues, the researchers detected the isoform-2 only in brain tissue, but not in other tissues; hence proposed that the shootin1 isoform-2 is a brain-specific protein^3^.

Here in this study, we showed for the first time that shootin1 is also expressed in PC12 cell line, which has a non-brain tissue origin; thus we revealed that shootin1 is not a protein specific to brain, but a protein specific to neuronal morphogenesis. PC12 is a cell line derived from rat adrenal medulla and when cultured in the presence of nerve growth factor (NGF), differentiates into sympathetic neuron-like cells morphologically and functionally^8^. Thus, PC12 cell line provides an exceptional experimental model for studying molecular processes associated with neuronal differentiation and morphogenesis. The ability of PC12 cells to differentiate in response to NGF allows for comparisons between pre- and post-differentiation dynamics. In addition to showing that both isoforms of shootin1 are expressed in PC12 cells, we elucidated that the activation of PI3K/Akt pathway is required for the production of isoform-2 variant. Consequently, we demonstrated that during neuronal differentiation of PC12 cells, the NGF-induced expression of shootin1 isoform-2 is accompanied by alternative splicing of shootin1 precursor mRNA.

## Results

### NGF induces expression of shootin1 isoform-2 protein during neuronal differentiation

**Figure 1:**
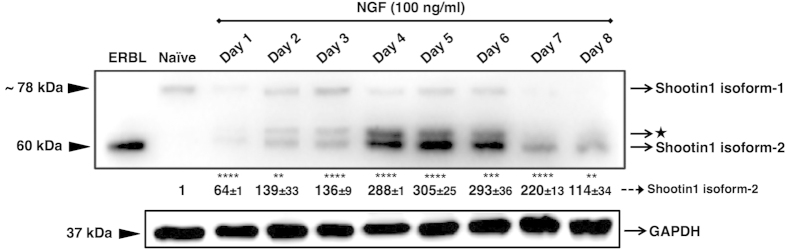
Western blot and densitometric quantification of shootin1 protein in PC12 cell lysates. Cells were incubated in the presence or absence of 100 ng/ml NGF for 8 days in differentiation medium. The presented immunoblot is a representative of four independent experiments with similar results. The relative intensities of shootin1 isoform-2 bands are shown under the immunoblot after normalization for the levels of GAPDH. The antibody used in this study recognizes residues surrounding the Lys450 of shootin1 (Cell Signaling Technology #3279). This conserved epitope is found in both shootin1 isoform-1 and isoform-2. ★ represents an unknown band, which may be a post-translationally modified form of the isoform-2. ERBL: Embryonic Rat Brain Lysate; Naïve: NGF-untreated. Values were expressed as mean ± SEM (n = 4). **P < 0.01, ***P < 0.001, ****P < 0.0001; Student’s *t*-test.

We investigated the expressional status of shootin1 during neuronal differentiation of PC12 cells using western blotting, and found that the isoform-2, which was originally described as a brain-specific protein, was not expressed in naïve (not treated with NGF) PC12 cells, whereas following NGF stimulation, cells began to express the isoform-2 at readily detectable levels (Fig. 1), in accordance with the obtaining of the neuronal phenotype (Supplementary Figure S1). Moreover, approximately after six days of persistent NGF stimulation, cells showed a significant decline in the expression levels of both shootin1 isoforms (Fig. 1).

We showed that only the isoform-1 is constitutively expressed in PC12 cells, whereas NGF-treated cells express both isoforms, and both of them contain the same epitope recognized by the antibody, according to the bioinformatics databases and information from antibody manufacturer (Fig. 1).

### Repressing shootin1 expression inhibits neurite outgrowth

**Figure 2:**
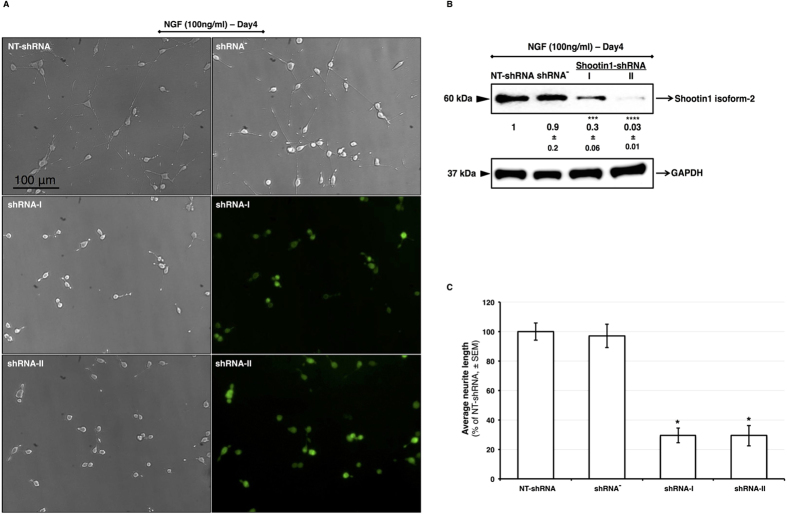
shRNA-mediated suppression of shootin1 expression resulted in a delay in neurite outgrowth. (**A**) Phase contrast and fluorescent images of stably transfected cells with shootin1 shRNA-I, shRNA-II and NT-shRNA (non-target) (**B**) Western blot analysis of shootin1 protein knockdown in PC12 cells transfected with shootin1 shRNA-I, shRNA-II, NT-shRNA and untransfected cells. Cell images were captured and cell lysates were collected after 4 days of NGF stimulation, separated on SDS-PAGE gels, and subjected to western blot analyses. (**C**) Neurite length analysis of untransfected or transfected cells with NT-shRNA, shRNA-I and shRNA-II. Images of 100–120 neurons per sample group were assessed. Values were expressed as mean ± SEM (n = 3). *P < 0.05, ***P < 0.001, ****P < 0.0001; Student’s *t*-test.

The functional role of shootin1 in PC12 cells was assessed using RNAi system. To determine whether shootin1 expression is required for the NGF-induced neurite extension, we constructed a PC12 cell line that stably expresses GFP as an expression marker and an shRNA targeting shootin1 mRNA, and evaluated cell morphology after NGF stimulation of the cells. Untransfected PC12 cells initiated neuritogenesis upon stimulation with NGF, whereas in transfected cells, the NGF-induced neurite extension was significantly hindered, in agreement with the results of Toriyama *et al.* (Fig. 2A). The interfering effect of the shRNA demonstrated with the microscopy images was in correlation with the reduced expression level of shootin1 isoform-2, as evidenced by western blotting (Fig. 2B). Neurite length measurements were calculated as the distance from the edge of the cell body to the tip of the growth cone to confirm that the inhibition of shootin1 expression by shRNAs impairs neurite outgrowth (Fig. 2C).

### Expression of shootin1 isoform-2 is regulated by PI3K/Akt pathway

**Figure 3:**
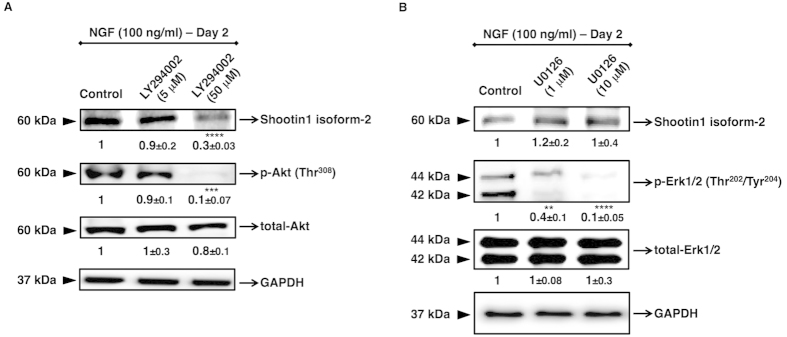
Effects of PI3K and Ras/MAPK inhibitors on NGF-induced activation of shootin1 in PC12 cells. Cells were pre-treated either with PI3K inhibitor LY294002 (5 and 50 μM), or with Ras/MAPK inhibitor U0126 (1 and 10 μM) for 2 h followed by treatment with NGF (100 ng/ml) for 48 h. Cells were harvested 48 h after treatments and lysates were prepared to determine the levels of the isoform-2 of shootin1 protein. (**A**) Densitometric quantification of isoform-2 of shootin1 protein or phosphorylated and total Akt protein levels after NGF stimulation of cells pre-treated with PI3K inhibitor. (**B**) Densitometric quantification of isoform-2 of shootin1 protein or phosphorylated and total Erk1/2 protein levels after NGF stimulation of cells pre-treated with Ras/MAPK inhibitor. The presented immunoblots are representative of three independent experiments with similar results. Relative intensities of shootin1 isoform-2, total-Akt, p-Akt, total-Erk1/2 and p-Erk1/2 are shown under immunoblots after normalization for the levels of GAPDH. Values were expressed as mean ± SEM (n = 3). **P < 0.01, ***P < 0.001, ****P < 0.0001; Student’s *t*-test.

NGF, which initiates the neuronal differentiation of PC12 cells, triggers intracellular signalling pathways such as Ras/MAPK and PI3K/Akt^9^. These pathways activate intracellular mediator molecules and transcription factors that execute neuronal differentiation processes in PC12 cell line, which is also mainly characterized by the expression of neuronal marker proteins^10^. We hypothesized that the expression of shootin1 isoform-2 is likely to be induced in cooperation with the NGF-induced kinase pathways that mediates neurite outgrowth during neuronal polarization. Thus, in this study, by using specific pharmacological inhibitors, Ras/MAPK and PI3K/Akt pathways were selectively blocked. PC12 cells were pre-treated with inhibitors for two hours according to the datasheets provided by the manufacturer, and then cultured in the presence of NGF for 2 days, considering the apparent long-term cytotoxic effects of the inhibitors. We evaluated the effect of LY294002, which is a selective pharmacological inhibitor for PI3K, and U0126, which is a selective pharmacological inhibitor for MEK1/2. As a result, it was revealed that shootin1 isoform-2 expression was mainly activated via PI3K/Akt pathway (Fig. 3A). In contrast, inhibition of Ras/MAPK pathway did not block the shootin1 expression (Fig. 3B).

### Alternative splicing of shootin1 pre-mRNA is regulated by NGF in PC12 cells

Isoform-1 of shootin1 protein is constitutively expressed in PC12 cells, while NGF-treated cultures express both the isoform-1 and isoform-2. In order to confirm that the shootin1 gene is constitutively open, we examined the methylation status of the CpG island in its promoter region in presence or absence of NGF, using bisulfite sequence analysis (Supplementary Materials and Methods). Although the promoter region of the shootin1 gene has not been fully characterized yet, the fact that its 5′ upstream region contains a typical CpG island susceptible to methylation, we evaluated the region on the DNA sequence that is 365 bases upstream (−377/−13) of the transcription start site of shootin1 gene. Bisulfite sequence analysis revealed that neither PC12 cells stimulated with NGF for 2 days nor naïve PC12 cells were differentially methylated (Supplementary Figure S2), which is in concordance with the constitutive expression of shootin1 isoform-1 (Supplementary Figure S1A).

**Figure 4:**
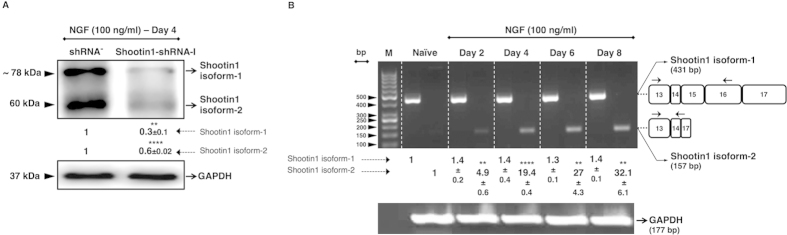
Isoform-1 and isoform-2 of shootin1 are differentially expressed in PC12 cells. (**A**) Western blot and densitometric quantification of protein knockdown of the isoform-1 and isoform-2 of shootin1 in PC12 cells transfected with shootin1-shRNA. Cell lysates were collected 4 days after NGF stimulation. (**B**) Isoform-specific RT-PCR results for the isoform-1 and isoform-2 of shootin1 transcripts, which shows an NGF-dependent transcription pattern for the isoform-2. Total RNA was isolated from PC12 cells at different days of NGF stimulation and subjected to RT-PCR, then agarose gel electrophoresis. Numbered white boxes at right panel represent the alternative exons within the isoform-1 and isoform-2. Small arrows above the white boxes represent the locations of isoform-specific oligonucleotide primers. Relative intensities of shootin1 isoforms both for the immunoblot and gel electrophoresis are shown under the images after normalization to the levels of GAPDH. M: Marker; Naïve: NGF-untreated; bp: base pair. Values were expressed as mean ± SEM (n = 3). **P < 0.01, ****P < 0.0001; Student’s *t*-test.

By using an shRNA targeting a common sequence in both variants of shootin1, we demonstrated that the expression of both isoforms of shootin1 was significantly suppressed on the protein level (Fig. 4A). Furthermore, in addition to the western blot results, we performed RT-PCR in order to determine the specific actions of NGF on shootin1 mRNA expression in PC12 cells for 8 days. Our isoform specific RT-PCR data proved that the isoform-1 and the isoform-2 of shootin1 were differentially expressed in PC12 cells upon NGF stimulation (Fig. 4B).

**Figure 5:**
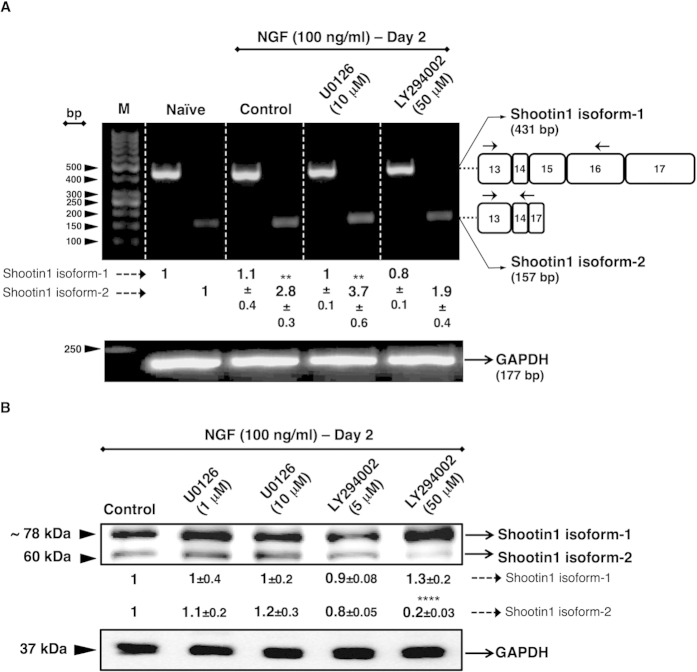
Effect of NGF-PI3K/Akt axis on alternative splicing of shootin1 in PC12 cells. (**A**) Isoform-specific RT-PCR results for the isoform-1 and isoform-2 of shootin1 transcripts after pre-treatment of the cells either with PI3K inhibitor LY294002 (50 μM), or with Ras/MAPK inhibitor U0126 (10 μM) for 2 h followed by treatment with NGF (100 ng/ml) for 48 h. Total RNA was isolated from the cells after 2 days of NGF stimulation and subjected to RT-PCR, then agarose gel electrophoresis. Numbered white boxes at right panel represent the alternative exons within the isoform-1 and isoform-2. Small arrows above the white boxes represent the locations of isoform-specific oligonucleotide primers. (**B**) Western blot results of the effects of PI3K inhibitor LY294002 (5 and 50 μM), or Ras/MAPK inhibitor U0126 (1 and 10 μM). PC12 cells were separately pre-treated with one of the inhibitors for 2 h followed by treatment with NGF (100 ng/ml) for 48 h. Cells were harvested 2 days after treatments and lysates were prepared to determine the levels of shootin1 protein isoforms. Relative intensities of shootin1 isoforms both for the immunoblot and gel electrophoresis are shown under the images after normalization to the levels of GAPDH. M: Marker; Naïve: NGF-untreated; bp: base pair. Values were expressed as mean ± SEM (n = 3). **P < 0.01, ****P < 0.0001; Student’s *t*-test.

**Figure 6:**
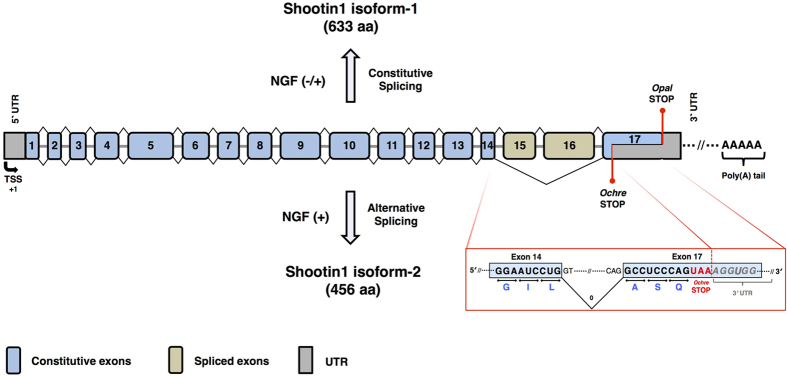
Splicing pattern for the generation of shootin1 isoform-1 and isoform-2. Shootin1 isoform-1 is constitutively spliced both in the presence and absence of NGF, and this isoform consists of exons 1–17. Generation of the isoform-2 is led by NGF-induced alternative splicing and involves the exclusion of exon 15 and 16 from the mRNA, which results in the formation of a premature termination codon within the 5′ region of the exon 17. The inset depicts the intron phase that leads to formation of the premature termination codon (*Ochre STOP*) during the generation of the isoform-2. TSS: Transcription Start Site.

The long mRNA transcript (3581 bases) encodes the isoform-1 (633 aa) while the short mRNA transcript (1371 bases) encodes the isoform-2 protein (456 aa). In order to determine whether the observed expression pattern of shootin1 isoforms is a consequence of alternative splicing, we performed RT-PCR by using oligonucleotide primers that are specifically complementary to regions individually located on the short and the long transcripts. We found that the isoform-1 exhibited a transcription pattern in an NGF-independent manner, whereas the isoform-2 displayed a transcription pattern that is strictly dependent on NGF stimulation. As a result, we concluded that this expression pattern represents the constitutive and NGF-induced alternative splicing of shootin1 pre-mRNA, which leads to the generation of the isoform-1 and isoform-2, respectively (Fig. 4B). Together with the results showing that the expression of shootin1 isoform-2 is strictly dependent on NGF stimulation and that it is regulated by PI3K pathway, we concluded that NGF-PI3K/Akt is the main axis that regulates the expression of this isoform. We confirmed the regulatory effect of NGF-PI3K/Akt axis both on the mRNA and protein levels of shootin1 isoform-2, by using PI3K inhibitor, LY294002 (Fig. 5A,B and Supplementary Figure S3). We also evaluated the effect of MEK1/2 inhibitor, U0126, however U0126 did not exhibit a significant difference neither on mRNA nor on protein level of the isoform-2 of shootin1 (Fig. 5A,B). As a conclusion, taken together with our results and the sequence information related to shootin1, we suggest a splicing pattern for the generation of the isoform-2 that involves the exclusion of exon 15 and 16 from the mRNA, which results in the formation of a premature termination codon (PTC) within the 5′ region of the exon 17 (Fig. 6).

These results suggest that NGF leads to an increase in the amount of the isoform-2 by means of alternative splicing in association with the neurite formation and differentiation of these cells. To our knowledge, these are the first experimental data demonstrating an NGF-induced alternative splicing during neurite formation and the first description of the splicing pattern of shootin1 pre-mRNA.

## Discussion

In this study, we firstly investigated that whether shootin1 protein is expressed in PC12 cells in presence or absence of NGF-stimulation. NGF-induced neuronal differentiation of PC12 cells provides an ideal model for studying processes associated with neuronal differentiation in a controllable manner^11^. The differentiation of rat PC12 cells into sympathetic neurons in response to NGF exhibits striking morphological and biochemical changes, which is evidenced by alterations in the transcriptional activity of various genes associated with neurite outgrowth, neurotransmitter synthesis and expression of neuron-specific markers^12^. Shootin1 protein was originally proposed as a novel, brain-specific protein by Toriyama *et al.* Interestingly, we found that shootin1 is expressed in non-brain-derived PC12 cells after differentiating into neuronal cells in response to NGF.

In their seminal work, Toriyama *et al.* reported that immunoblot analysis of various rat tissues detected shootin1 only in brains, but not in other tissues; moreover, they demonstrated that shootin1 has an essential role during the organization of axonal specification. Taking all together, they suggested that shootin1 is a brain-specific protein^3^. In agreement with these results, we showed that shootin1 is expressed in PC12 cells during neuronal differentiation, which suggests that shootin1 is a protein that has a fundamental role in neuritogenesis. Furthermore, we showed that the stably-transfected PC12 cells expressing shRNA specific to shootin1 mRNA, exhibited significantly decreased protein expression and remarkably obstructed neurite elongation; these results are consistent with the findings of Toriyama *et al.,* which were demonstrated in rat hippocampal neurons^3^.

In this report, we describe a novel signalling connection in which NGF-dependent activation of PI3K leads to stimulation of shootin1 protein expression to promote neurite elongation in PC12 cells. We showed that shootin1 expression is induced by the stimulation of NGF and suppressed by LY294002 that inhibits NGF-activated PI3K pathway, indicating that PI3K pathway is critical for NGF-mediated shootin1 expression in these cells. The action of NGF on shootin1 expression appears to be independent of Ras/MAPK, the other main pathway activated by NGF^9^, since the inhibition of the pathway by U0126 did not disrupt shootin1 expression in presence of NGF. Previous results on the interaction between shootin1 protein and PI3K suggested that shootin1 protein functions upstream of PI3K, and hence organizes the intracellular localization of PI3K activity^3^,^4^. Taking into account the earlier findings together with our presented results, we propose a novel feedback loop model in which shootin1 expression is mediated via PI3K pathway and consequent dynamics of shootin1 organizes the intracellular localization of PI3K.

Alternative splicing is a ubiquitous mechanism that allows diverse protein isoforms to be originated from one genetic template. It plays a critical role in the expression of a wide variety of proteins in the nervous system, including Trk receptors and neural cell adhesion molecules^13^,^14^. Alternative splicing of the pre-mRNA transcript of the rat shootin1 gene results in the production of distinct mRNAs encoding the isoform-1 or the isoform-2 of shootin1. The brain-specific isoform-2 mRNA predominates in only differentiated PC12 cells while the isoform-1 mRNA appears both in naïve and differentiated cells. These observations led us to propose a model, which suggests that neuro-developmental regulation of the pre-mRNA processing is used to increase the diversity of shootin1 gene expression.

In this study, we demonstrated that there is an expressional disparity between the mRNA levels of shootin1 isoform-1 and isoform-2 and their protein levels, in NGF-induced cells for 8 days. The mRNA expression of the isoform-1 displays a consistent profile, and the mRNA expression of the isoform-2 exhibits a constant up-regulation following NGF stimulation; on the other hand, protein expression levels of both the isoform-1 and isoform-2 exhibit a significant down-regulation after 6 days of NGF stimulation. A hypothetical mechanism that leads to this transcript/protein expression decoupling on the 6th day of NGF stimulation may be related to the establishment of the neuronal phenotype by the 6th day^15^, so that, along with the achievement of the maximal extent of neurite outgrowth, several feedback mechanisms may be triggered to interfere with the translation machinery or to activate the proteosomal degradation of the protein without affecting the mRNA levels^16^. Ubiquitin-mediated proteolysis plays an important role in coordinating neuronal differentiation, migration and maturation. Several studies showed that the ubiquitin-mediated proteolysis of specific axonal proteins has a critical regulatory role on generation of a polarized neuron and specification of a single neurite into an axon^17^,^18^. In parallel, a proteomic study performed in murine tissues revealed that shootin1 protein is post-translationally ubiquitinated at Lys89 and Lys244 residues^19^. Moreover, proteomic analyses revealed a divergent pattern of ubiquitylation sites in murine tissues^19^. Therefore, we suggest that the time-dependent down-regulation of the shootin1 protein isoforms may be regulated by ubiquitylation by the end of neurogenesis.

Additionally, our data provide the first information on the molecular mechanisms regulating the expression of shootin1 gene: NGF activates the TrkA receptor tyrosine kinase, which results in the activation of a number of intracellular signalling pathways, including PI3K that induces protein expression of isoform-2 of shootin1 only during NGF-mediated differentiation of PC12 cells. Thus we showed that the activation of PI3K/Akt pathway has a major role in the formation of the splicing variants of shootin1.

Several studies showed that alternative splice sites located either in exons or introns are preferentially selected by trans-acting splicing factors such as serine-arginine rich proteins (SRs) or heterogeneous nuclear ribonucleoproteins (hnRNPs) depending on the sequence motifs of cis-regulatory elements (exonic/intronic splicing enhancers/silencers)^20^,^21^. In response to extracellular stimuli, PI3K-mediated phosphorylation of Akt directly or indirectly (through serine/arginine-rich protein-specific kinases; SRPK1/2) phosphorylates SR proteins resulting in their translocation into the nucleus where they bind to corresponding pre-mRNAs; besides, it is also known that Akt can phosphorylate hnRNPs as well, for further action^22^,^23^,^24^. Such a process allows a rapid response to varying environmental conditions as alternative variants can be generated without extra *de novo* cycles of mRNA synthesis. Hence, we suggest that Akt, as the downstream effector of PI3K, seems to modulate alternative splicing of shootin1 pre-mRNA, and the present data suggest a direct action of this kinase on trans-acting splicing factors.

Shootin1 appears to be a neuron-specific protein and it is noteworthy that the tissue-specific expression of shootin1 might be strictly regulated by several mechanisms. It has been also known that mRNA maturation has a number of quality control mechanisms for the safety of the information carried by mRNAs. One of these, an alternative splicing-related mechanism, is nonsense-mediated mRNA decay (NMD) system that recognizes and degrades aberrant mRNAs with truncated open reading frames by means of the formation of a PTC either at internal or terminal exons^25^. Thus, it leads to rapid decay of mRNAs that are aberrantly spliced, in order to eliminate the synthesis of non-functional and/or potentially detrimental truncated proteins^26^,^27^. According to our RT-PCR data, the neuron-specific isoform-2 of shootin1 is slightly being transcribed but not translated in naïve cells. In parallel, presented splicing pattern in Fig. 6 shows that splicing of isoform-2 from the pre-mRNA generates a PTC at the terminal exon. These data suggest that the mRNA of shootin1 isoform-2 may be degraded by NMD system in the absence of NGF; upon NGF stimulation, NMD mechanisms may become inhibited via as yet unknown mechanisms.

In conclusion, here we report that NGF modulates alternative splicing of shootin1 precursor mRNA in PC12 cells via activating PI3K/Akt pathway. These results expand the understanding of both the actions of NGF and the regulation of shootin1 gene expression, which also indicates that similar mechanisms may not only be important in governing the expression of shootin1 gene *in vivo*, but also in expression of other neuron-specific genes. Furthermore, this study clearly demonstrates that shootin1 isoform-2 functions as a neurite formation mediator in PC12 cells, and acts as a physical linkage between NGF-stimulation and neuronal morphogenesis. On the other hand, there is currently no experimental data on the function and dynamics of the shootin1 isoform-1 and the issue of exactly what biological role it has is yet unclear. However, although it shares the first 14 exons with the isoform-2, shootin1 isoform-1 protein has 177 extra amino acids. Moreover, isofom-1 seems to have different domain architecture than that of the isoform-2 protein, which is named as Smc (Chromosome segregation ATPases; NCBI Conserved Domain Database-CDD; Location: 7 → 344; Accession: COG1196). This putative domain that the isoform-1 possesses is thought to be responsible for cell cycle control, cell division and chromosome partitioning. Besides, proteomic analyses show that the additional protein regions within the isoform-1 contain a high number post-translational modification regions (PhosphoSitePlus^®^ for shootin1). Further clarification of the function and the dynamics of shootin1 isoform-1 in neuronal survival and differentiation will undoubtedly illuminate several other mechanisms that have not yet been elucidated. Detailed molecular mechanisms involved in neuronal polarity formation remain important questions for future analyses and NGF-induced changes in shootin1 gene expression in PC12 cells may have a leading role in future studies. Further studies are necessary to understand the exact molecular mechanisms of NGF/TrkA/PI3K axis, which results in the alternative splicing of shootin1 pre-mRNA.

## Methods

### Materials

PC12 cells were obtained from American Type Culture Collection (Manassas, VA, USA) and embryonic rat brain lysate was kindly provided by Bryon Ricketts (Cell Signaling Technology “CST”; The Netherlands). Fetal bovine serum (FBS), horse serum, RPMI medium were purchased from HyClone (Logan, UT, USA). Mouse nerve growth factor 2.5S (2.5S mNGF) was obtained from Promega (Madison, WI, USA). Collagen Type IV from human placenta was purchased from Sigma-Aldrich (Steinheim, Germany). Rabbit mono- or poly-clonal antibodies to shootin1, ERK1/2, p-ERK1/2 (Thr202/Tyr204), Akt, p-Akt (Thr308) and GAPDH were obtained from CST (Danvers, MA, USA). Pharmacological inhibitors, U0126 (MEK1/2 inhibitor) and LY294002 (PI3 kinase-dependent Akt phosphorylation inhibitor) were purchased from CST (Danvers, MA, USA).

### Cell culture

Cells were maintained *in vitro* using RPMI medium supplemented with 10% heat-inactivated horse serum, 5% FBS, 2 mM L-glutamine and 1% penicillin-streptomycin (complete medium). Cells were cultured in a humidified atmosphere of 5% CO2 and 95% air at 37 °C. This cell line responds reversibly to NGF and differentiate into neuronal phenotype when plated on collagen type IV coated culture flasks in RPMI medium supplemented with 1% heat-inactivated horse serum, 2 mM L-glutamine, 1% penicillin-streptomycin and 100 ng/ml NGF (differentiation medium).

### Inhibitor treatment

PC12 cells were pre-incubated for 2 h in the presence of U0126 (MEK1/2 inhibitor; 10 μM) or LY294002 (PI3K inhibitor; 50 μM) before addition of NGF (100 ng/ml) according to the manufacturer’s recommendation. After treatment of cells pre-incubated with inhibitors for 2 h and with NGF for 48 h, cells were either lysed in RIPA cell lysis buffer (CST, Danvers, MA, USA) or with High Pure RNA Kit (Roche; Mannheim, Germany) for western blotting and RT-PCR, respectively.

### Gene silencing and generation of stable cell line

Inhibition of shootin1 expression via RNAi was performed. Briefly, two different plasmids (psiRNA-h7SK-GFP:ZEO-Shootin1-I and -II; Invivogen, San Diego, CA, USA) carrying a fluorescent reporter (GFP) and a short-hairpin RNA targeting two different regions of rat shootin1 mRNA, and a negative control shRNA targeting LacZ mRNA (NT-shRNA; shRNA-LacZ) were introduced using Lipofectamine LTX & PLUS reagent (Invitrogen, Carlsbad, CA, USA). GFP was used to confirm the efficient delivery of shRNA plasmid into cells. To establish a stable cell line expressing shootin1 specific shRNA, PC12 cells (1 × 10^6^) were transfected with 5 μg of plasmid carrying the shRNA complementary to a region of shootin1 mRNA, using Lipofectamine LTX & PLUS reagent. One day after transfections, the cells were replated at a lower density and selected with 250 μg/ml Zeocin for 30 days. Individual colonies were isolated, grown up and tested by PCR for the incorporation of the plasmid into genomic DNA. shRNA sequences can be found as Supplementary Table S1. The efficiencies of the protein knockdown and neurite outgrowth were assessed by western blotting and fluorescence microscopy.

### Measurement of neurite length

Growing cells were plated on collagen type IV from human placenta pre-coated 60 mm dishes. Adhered cells then left untransfected or transfected with NT-shRNA, shRNA-I and shRNA-II, separately. Cells were then analyzed by phase contrast microscopy and counted for neurite outgrowth. For the length measurements, the neurites equal to or greater than a cell body in length were considered. The lengths were measured as the distance between the edge of a cell body and the tip of a growth cone. Only clearly visible cells were subjected to analysis to prevent inaccurate scoring. Images of 100–120 neurons per sample group were captured for each experiment. Neurite lengths were measured by using NeuronJ (ImageJ plug-in; NIH, Bethesda, MD, USA). The average neurite length was obtained by dividing the total neurite length by the number of cells and expressed as % control to yield the average neurite length per sample group.

### Western blotting

For protein expression analyses, PC12 cells cultured in 60 mm petri-dishes (Sarstedt, Nürnbrecht, Germany) were lysed in 100 μl of RIPA buffer supplemented with 1 mM PMSF (Roche Diagnostics, Mannheim, Germany). Protein concentrations were determined using the BCA protein assay (Pierce, Rockford, IL, USA). Protein lysates (20 μg) were heated for 5 min at 94 °C in Laemmli sample buffer containing 5% β-mercaptoethanol and then loaded on 4–15% Tris-glycine SDS-PAGE gels, then transferred electrophoretically onto polyvinylidene difluoride (PVDF; Bio-Rad, Hercules, CA, USA) membranes at 200 mAmp for 2 h. Membranes were blocked with 5% non-fat dry milk for 1 h and incubated overnight at 4 °C with the antibodies specific to Shootin1, ERK1/2, p-ERK1/2 (Thr202/Tyr204), Akt, p-Akt (Thr308) and GAPDH (CST, Danvers, MA, USA). Protein bands were detected with horseradish peroxidase-conjugated anti-rabbit secondary antibodies (CST; Danvers, MA, USA) and visualized by West-Femto ECL reagents (Pierce; Rockford, IL, USA). Chemiluminescent signals of immunoblots were documented using Gel Logic 2200 Pro (Carestream Health; Rochester, NY, USA). The net intensity of specific proteins was quantified using ImageJ (NIH, Bethesda, MD, USA).

### Reverse Transcriptase-Polymerase Chain Reaction (RT-PCR)

For the determination of splicing variants of shootin1 mRNA, total RNA was isolated from PC12 cells using a commercially available High Pure RNA Kit. RT-PCR was performed by using Transcriptor First Strand cDNA Synthesis kit (Roche; Mannheim, Germany). Briefly, 1 μg of total RNA reverse transcribed at 50 °C for 1 h using Transcriptor Reverse Transcriptase that has RNase H activity, and hexamer primers in a reaction volume of 20 μl. After cDNA synthesis, both for shootin1 and the housekeeping gene GAPDH, all cDNAs were amplified as follows: 35 cycles of 94 °C for 30 s; 55 °C for 30 s; and 72 °C for 45 s. The oligonucleotide primer sequences (Alpha DNA, Canada) used in this study can be found as Supplementary Table S1. Amplified products were resolved by 2% agarose gel electrophoresis, stained with ethidium bromide, and photographed under UV.

### Computational analysis

The promoter and cDNA sequences of shootin1 (RGD1311558; Gene ID: 292139) retrieved from Genbank (Rnor_5.0 r.104). Primers were designed with Primer3 software to detect the constitutive and alternative splicing products. To facilitate methylation detection, bisulfite PCR primers were designed by using the Methyl Primer Express Software v1.0 (Applied Biosystems). Comparative analysis of shootin1 protein variants for the regions that are recognized by shootin1 antibody in rat, were done using the BLASTp program.

### Statistical analysis

All the experiments were performed at least three times. Values were expressed as mean ± SEM. Statistical analysis was performed with unpaired Student’s *t*-test using GraphPad software. *P* values less than 0.05 were considered statistically significant (*P < 0.05; **P < 0.01; ***P < 0.001; ****P < 0.0001).

## Additional Information

**How to cite this article**: Ergin, V. *et al.* Regulation of Shootin1 Gene Expression Involves NGF-induced Alternative Splicing during Neuronal Differentiation of Pc12 Cells. *Sci. Rep.* **5**, 17931; doi: 10.1038/srep17931 (2015).

## Supplementary Material

Supplementary Information
