# Supplementary material for: Regulation of Shootin1 Gene Expression Involves NGF-induced Alternative Splicing during Neuronal Differentiation of PC12 Cells
Source: Sci Rep. 2015 Dec 9;5:17931. doi: 10.1038/srep17931 (PMC4673418; doi:10.1038/srep17931)
Supplement: Supplementary Information [file srep17931-s1.pdf]

SUPPLEMENTARY INFORMATION

Supplementary Figure S1 – S2 – S3
Supplementary Materials and Methods
Supplementary Table S1

REGULATION OF SHOOTIN1 GENE EXPRESSION INVOLVES NGF-INDUCED ALTERNATIVE SPLICING DURING NEURONAL DIFFERENTIATION OF PC12 CELLS

Volkan Ergin[1, 2, †, *], Mutlu Erdogan[3, 4, †], Adnan Menevse[1]

[1] Department of Medical Biology & Genetics, Faculty of Medicine, Gazi University, 06560 Ankara, Turkey

[2] Current address: Department of Medical Biology, Faculty of Medicine, Erzincan University, 24030 Erzincan, Turkey

[3] UNAM - Institute of Materials Science and Nanotechnology, Bilkent University, 06800 Ankara, Turkey

[4] Current address: Max Planck Institute of Neurobiology, 82152 Martinsried, Germany

[*] Correspondence and requests for materials should be addressed to V.E. (vergin@erzincan.edu.tr)

[†] These authors contributed equally to this work.

A

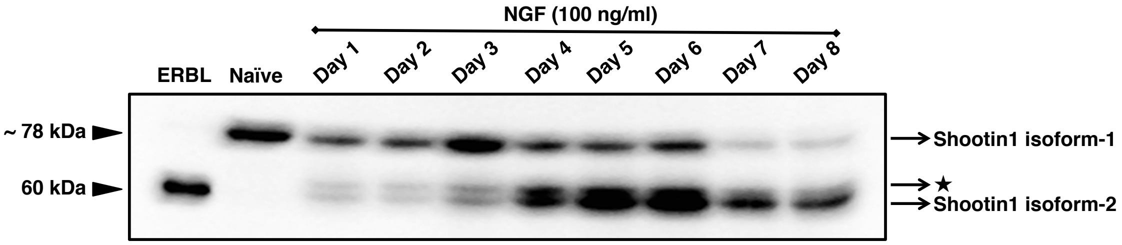
NGF (100 ng/ml)
ERBL
Naïve
Day 1
Day 2
Day 3
Day 4
Day 5
Day 6
Day 7
Day 8
~ 78 kDa
60 kDa
Shootin1 isoform-1
★
Shootin1 isoform-2

B

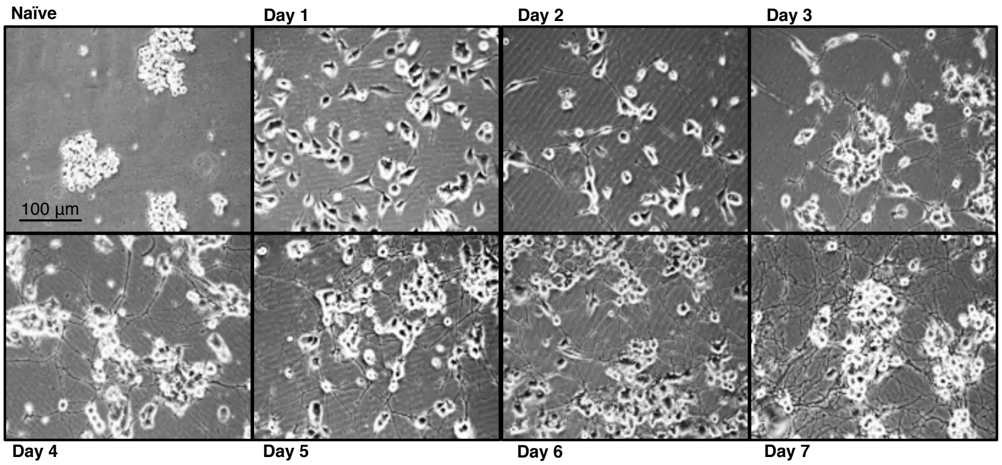
Naïve
Day 1
Day 2
Day 3
100 µm
Day 4
Day 5
Day 6
Day 7

C

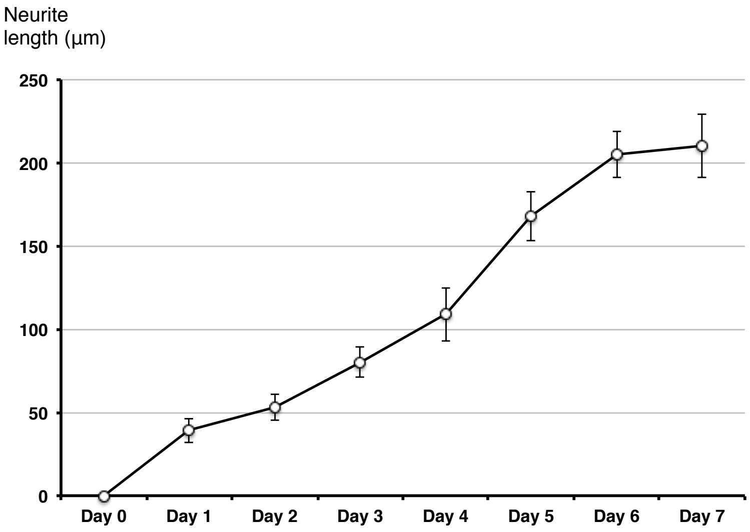
Neurite
length (µm)
250
200
150
100
50
0
Day 0
Day 1
Day 2
Day 3
Day 4
Day 5
Day 6
Day 7

Supplementary Figure S1. The isoform-1 of shootin1 is constitutively expressed in PC12 cells, whereas isoform-2 is expressed during NGF-induced differentiation of PC12 cells. A) Western blot image of shootin1 protein in PC12 cell lysates. Cells were incubated in the presence or absence of 100 ng/ml NGF for 8 days in differentiation medium. The antibody used in this study recognizes surrounding Lys^{450} of shootin1 (Cell Signaling Technology #3279). This conserved epitope is found in both shootin1 isoform-1 and isoform-2. ★ represents an unknown band, which may be a post-translationally modified form of the isoform-2. ERBL: Embryonic Rat Brain Lysate; Naïve: NGF-untreated. **B)** Morphological changes in PC12 cells during 7 days of NGF stimulation. **C)** Neurite length analysis of PC12 cells during 7 days of NGF stimulation. Values were expressed as mean ± SEM (n=4).

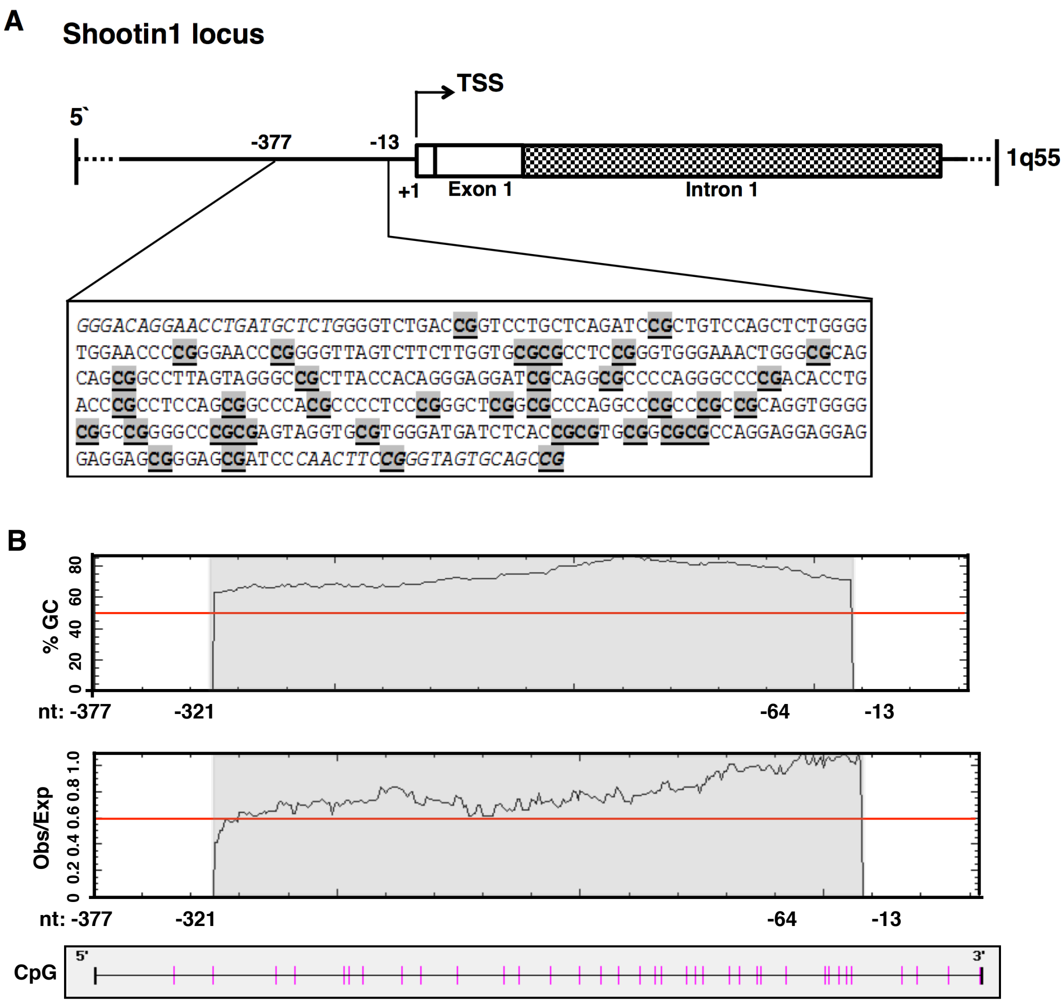
A
Shootin1 locus
TSS
5`
-377
-13
+1
Exon 1
Intron 1
1q55
GGGACAGGAACCTGATGCTCTGGGGTCTGACCGGTCCTGCTCAGATCCGCTGTCCAGCTCTGGGG
TGGAACCCCGGGAACCCGGGGTTAGTCTTCTTGGTGCGCGCCTCCGGGTGGGAAACTGGGCGCAG
CAGCGGCCTTAGTAGGGCCGCTTACCACAGGGAGGATCGCAGGCGCCCCAGGGCCCCGACACCTG
ACCCGCCTCCAGCGGCCCACGCCCCTCCCGGGCTCGGCGCCCAGGCCCGCCCCGCCGCAGGTGGGG
CGGCCGGGGCCCGCGAGTAGGTGCGTGGGATGATCTCACCGCGTGCGGCGCGCCAGGAGGAGGAG
GAGGAGCGGGAGCGATCCCAACTTCCGGGTAGTGCAGCCG
B
% GC
0 20 40 60 80
nt: -377
-321
-64
-13
Obs/Exp
0 0.2 0.4 0.6 0.8 1.0
nt: -377
-321
-64
-13
CpG
5'
3'

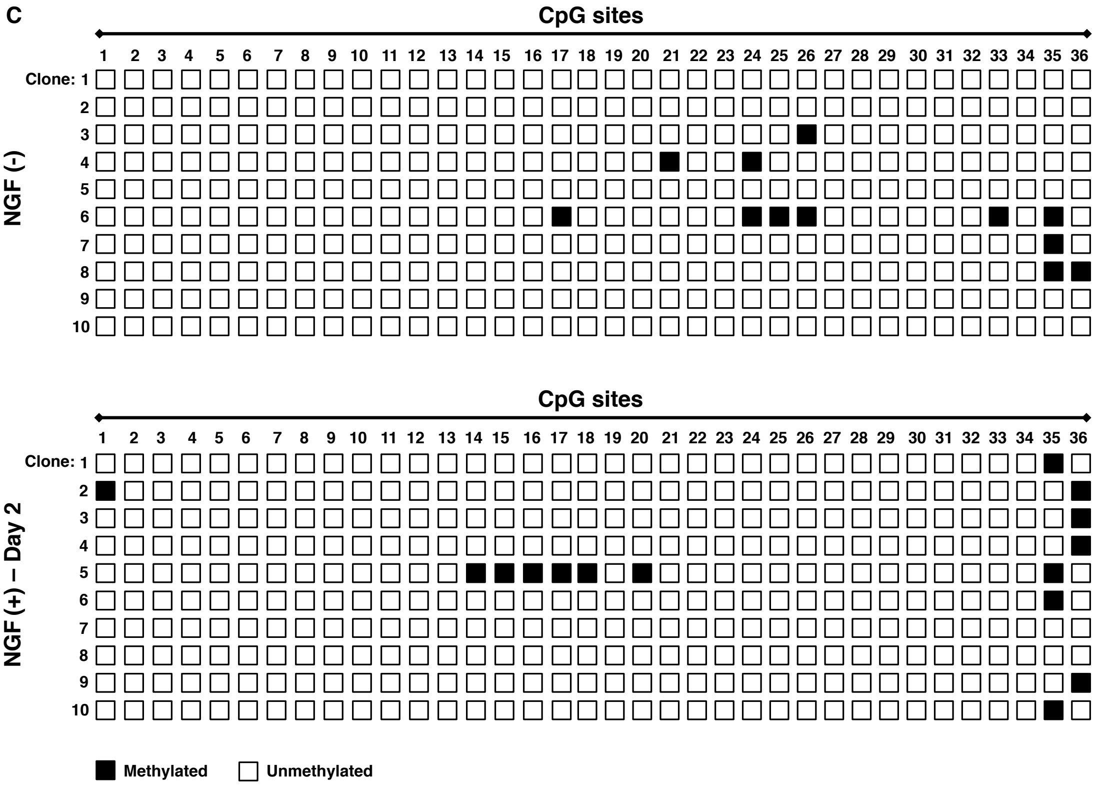

Supplementary Figure S2. Bisulfite sequence analysis of undifferentiated and differentiated PC12 cells. Methylation status of the CpG island in presence or absence of NGF, using bisulfite sequence analysis. **A)** Distribution of CpG dinucleotides within the 5` upstream region of shootin1 gene. **B)** To predict the potential CpG islands associated with the shootin1 gene, EMBOSS CpGPlot tool with standard parameters (>200 bp, G+C content > 50%, observed/expected CpG ratio > 0.6) was used. The program identified 36 potential CpG sites within the 5` upstream region of shootin1 gene, reaching from −321 to −64 bp in rat. Then, a region on the DNA sequence that is 365 bases upstream (−377 / −13) of the transcription start site was experimentally evaluated. **C)** Bisulfite sequence analysis, which allows a comprehensive examination of each methylated CpG site in a target sequence, revealed that neither PC12 cells stimulated with NGF for 2 days nor naïve PC12 cells were differentially methylated.

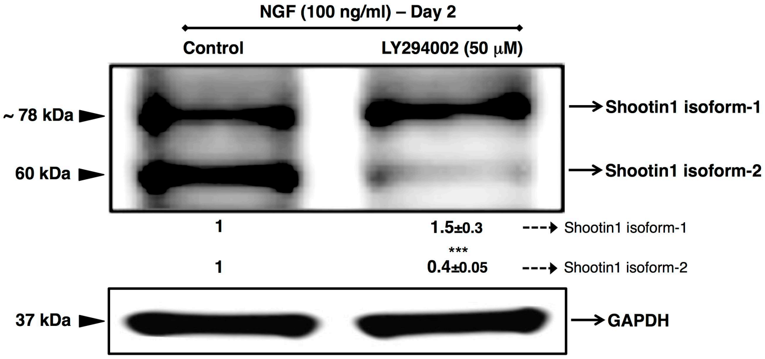

Supplementary Figure S3. NGF-PI3K/Akt axis regulates the expression of shootin1 isoform-2 in PC12 cells. Western blot and densitometric quantification of shootin1 protein in PC12 cell lysates. Cells were pre-treated with PI3K inhibitor LY294002 (50 μM) for 2 h followed by treatment with NGF (100 ng/ml) for 48 h, then were harvested and lysates were prepared to determine the protein levels of shootin1 isoforms using western blot analysis. Relative intensities of shootin1 isoform-1 and isoform-2 are shown under immunoblot after normalization for the levels of GAPDH. Values were expressed as mean ± SEM (n=3). ***$P < 0.001$, Student's *t*-test.

Supplementary Materials and Methods

Bisulfite sequencing. Genomic DNA was isolated from PC12 cells using the Genomic DNA Purification Kit (Fermentas; Vilnius, Lithuania) according to the manufacturer's directions, and it was digested with HindIII restriction endonuclease, which does not recognize our ROI. The fragmented DNA samples were modified with sodium bisulfite using the EZ DNA Methylation-Gold kit (Zymo Research, Orange, CA), as stated in the manufacturer's instructions. Briefly, the DNA was denatured with a dilution buffer containing 2 M NaOH and incubated overnight at 50 °C with C→T conversion reagent, followed by clean-up, desulphonation, and elution. The bisulfite-modified DNA was then immediately amplified using EpiMark Hot Start Taq DNA Polymerase (New England Biolabs; Beverly, MA, USA). PCR conditions for 5` upstream of shootin1 gene (-13 / -377) including 36 CpG sites were as follows: 35 cycles of 95 °C for 30 s; 58 °C for 45 s; and 68 °C for 30 s. The modified DNA was amplified with oligonucleotide primers capable of annealing both methylated and unmethylated alleles, and primers were designed against the selected region by using the Methyl Primer Express Software v1.0. (Applied Biosystems; Foster City, CA, USA). The primer pairs are shown in the Supplementary Table S1. Generated 365-bp long PCR products were separated on 2% agarose gels and visualized under UV illumination. The band of interest in the agarose gel was carefully excised with a razor blade under UV illumination, and then the amplicon was purified from the gel with Qiagen PCR purification kit (Chatsworth, CA, USA). To evaluate the methylation status of the promoter region of shootin1, PCR products were cloned into the T-tailed vector with pCR®2.1 using Original TA Cloning® Kit (Invitrogen; San Diego, CA, USA) and transformed into *E. coli* strain DH5alpha

according to the supplier's protocol. Transformants containing recombinant plasmids were selected using standard ampicillin antibiotic and X-Gal blue-white screening. At least 10 white clones were selected and then sequenced following colony PCR. Sequencing reactions were then performed using the M13 primers along with BigDye Terminator v3.1 Cycle Sequencing kit (Applied Biosystems; Foster City, CA, USA) before separation on an ABI 3100 DNA sequence analyzer. DNA sequence chromatograms were analyzed using FinchTV v.1.4 (Geospira Inc.; Seattle, WA, USA).

Supplementary Table S1. List of primers used for RT–PCR and bisulfite sequencing, and short hairpin sequences targeted to shootin1 mRNA and to non-target LacZ transcript.

**RT-PCR**				
**Gene name**	**Forward sequence (5`→3`)**	**Reverse sequence (5`→3`)**	**Amplicon (bp)**	**Reference**
Shootin1 isoform-1	CTGAAGAGGCAAGCAGTGGA	TCCAATTTACGGGGCCCTTC	431	This study
Shootin1 isoform-2	CTGAAGAGGCAAGCAGTGGA	ACTGGGAGGCCAGGATTC	157	This study
GAPDH	GTTACCAGGGCTGCCTTCTC	GATGGTGATGGGTTTCCCGT	177	This study
**Bisulfite Sequencing**				
**Gene name**	**Forward sequence (5`→3`)**	**Reverse sequence (5`→3`)**	**Amplicon (bp)**	**Reference**
Shootin1 (5`-upstream)	GGGATAGGAATTTGATGTTTTG	CRACTACACTACCCRAAAATTA	365	This study
**shRNAs**				
**Target gene**	**shRNA sequence**		**Position**	**Reference**
Shootin1shRNA-I	TGAAGCTGTTAAGAAACTGGActcttgaTCCAGTTTCTTAACAGCTTCA		+223 - +243	Toriyama et al. 2006
Shootin1 shRNA-II	GCAGAACCACCTTGAGATAGAtcaagagTCTATCTCAAGGTGGTTCTGC		+292- +312	This study
LacZshRNA	GTGACCAGCGAATACCTGTTctcttgaAACAGGTATTCGCTGGTCAC		N/A	Invivogen (psiRNA-h7SKGFPzeo Kit)

Nucleotide sequences of shootin1 gene and mRNA were provided by GenBank Rnor_5.0 r.104.
